# Inhibitory Effects of Syringic Acid on Endometrial Cancer Cell Growth and Migration and Its Synergistic Suppression with Doxorubicin

**DOI:** 10.3390/ph18111596

**Published:** 2025-10-22

**Authors:** Yi-Ting Kuo, Chi-Chang Chang, Yu Chang, Chin-Feng Hsuan, Tzu-Hsien Chang, Ya-Ling Chen, Hsin-Ya Houng, Yu-Chieh Su, Jer-Yiing Houng

**Affiliations:** 1 Graduate Institute of Medicine, College of Medicine, I-Shou University, Kaohsiung 824005, Taiwan; gazilla0403@gmail.com; 2 School of Medicine for International Students, College of Medicine, I-Shou University, Kaohsiung 824005, Taiwan; ed101779@edah.org.tw (C.-C.C.); changyu0510@gmail.com (Y.C.); 3 Department of Obstetrics & Gynecology, E-Da Hospital, I-Shou University, Kaohsiung 824005, Taiwan; 4 Department of Obstetrics & Gynecology, E-Da Dachang Hospital, I-Shou University, Kaohsiung 824005, Taiwan; andy3560133@gmail.com (T.-H.C.); igiolal2011@gmail.com (Y.-L.C.); nhy8381@gmail.com (H.-Y.H.); 5 School of Medicine, College of Medicine, I-Shou University, Kaohsiung 824005, Taiwan; ed102745@edah.org.tw; 6 Division of Cardiology, Department of Internal Medicine, E-Da Hospital, I-Shou University, Kaohsiung 824005, Taiwan; 7 Division of Cardiology, Department of Internal Medicine, E-Da Dachang Hospital, I-Shou University, Kaohsiung 824005, Taiwan; 8 Division of Hematology-Oncology, Department of Internal Medicine, E-Da Hospital, I-Shou University, Kaohsiung 824005, Taiwan; 9 Department of Nutrition, I-Shou University, Kaohsiung 824005, Taiwan

**Keywords:** syringic acid, endometrial cancer cells, human endometrial stromal cells, proliferation, migration

## Abstract

**Background/Objectives**: Endometrial cancer (EC), a malignancy arising from the uterine lining, is a leading gynecological cancer in developed countries. Syringic acid (SA), a naturally occurring phenolic compound, possesses various bioactivities including antioxidant, anti-inflammatory, chemoprotective, and anti-angiogenic properties. This study aimed to investigate the effects of SA on the proliferation and migration of RL95-2 EC cells, its protective role in normal endometrial stromal cells (HESCs), and the underlying molecular mechanisms. Furthermore, the potential synergistic anticancer effects of SA in combination with chemotherapeutic agents against EC were evaluated. **Methods**: Cell viability was assessed using nuclear fluorescence staining, the MTT assay, and clonogenic survival assay. Cell migration was evaluated through wound closure and Transwell migration assays. Gene expression levels were analyzed by the RT-PCR method. **Results**: SA significantly inhibited the proliferation of RL95-2 EC cells, with an IC_50_ value of 27.22 μM. Co-treatment with SA and the chemotherapeutic agent doxorubicin (Dox) demonstrated an additive inhibitory effect. Mechanistically, both SA and the SA-Dox combination induced apoptosis by upregulating the expression of *caspases-3*, *-8*, and *-9*, increasing the expression of pro-apoptotic genes (*Bax* and *Bad*), and downregulating anti-apoptotic genes (*Bcl-XL* and *Bcl-2*). Cell cycle analysis revealed the downregulation of *cyclin D* and the upregulation of tumor suppressors *p21* and *p27*, contributing to growth arrest. In addition, both SA and the combination treatment effectively suppressed cell migration by downregulating matrix metalloproteinases (*MMP*s) and *β-catenin*. SA treatment also induced the expression of pro-inflammatory cytokines (TNF-α, IL-6, IL-1β) and activated NF-κB signaling, leading to an elevated expression of inflammatory mediators such as *COX-2* and *iNOS*. Furthermore, SA promoted oxidative stress in RL95-2 cells by inhibiting the Nrf2 pathway and reducing the expression and activities of antioxidant enzymes including catalase, glutathione peroxidase, and superoxide dismutase, thereby enhancing reactive oxygen species (ROS) accumulation. In contrast, in lipopolysaccharide-stimulated HESC cells, SA attenuated inflammation and ROS generation, indicating its selective cytoprotective role in normal endometrial cells. **Conclusions**: SA may serve as a promising adjuvant candidate to enhance chemotherapeutic efficacy while protecting normal cells by mitigating inflammation and oxidative stress.

## 1. Introduction

Endometrial cancer (EC), arising from the endometrial lining of the uterus, is the 15th most common cancer globally and the sixth most prevalent among women. Although primarily affecting postmenopausal women, it can occur at any age. The global age-standardized incidence rate is approximately 8.4 per 100,000 women, and the incidence of EC has been steadily increasing, especially in developed countries [1]. Risk factors associated with EC include age, obesity, hormonal imbalances, physical inactivity, endometrial hyperplasia, excessive exogenous estrogen, insulin resistance, a history of breast or ovarian cancer, the use of hormone replacement therapy, and genetic predispositions [2,3].

While early diagnosis and surgical treatment have advanced, effective management of advanced or recurrent EC remains a considerable clinical challenge. Standard chemotherapeutic regimens, including cisplatin (Cis), doxorubicin (Dox), paclitaxel (PTX), and topotecan (Top), are limited by several factors: notable off-target cytotoxicity, poor tumor selectivity, intolerable side effects, and the emergence of multi-drug resistance. These limitations are particularly problematic in metastatic or recurrent presentations. Adverse events, such as severe gastrointestinal dysregulation, intractable nausea, emesis, mucosal degradation, alopecia, nephrotoxicity, cardiotoxicity, and bone marrow suppression, exert significant physiological and psychological burdens on patients. Collectively, these complications contribute to poor prognosis and reduced quality of life in late-stage or recurrent EC [4,5].

Natural compounds derived from plant sources, such as flavonoids, polyphenols, terpenes, and alkaloids, have demonstrated significant anticancer potential in both in vitro and in vivo models, particularly against EC cells. These natural derivatives exhibit antioxidative, anti-inflammatory, pro-apoptotic, and antimetastatic effects. Compared with synthetic drugs, phytochemicals are associated with lower toxicity, fewer adverse reactions, and better patient tolerability. Importantly, their ability to modulate multiple oncogenic pathways and immune responses as well as reduce resistance to chemotherapy underscores their therapeutic relevance [6,7]. A central mechanism involves the manipulation of reactive oxygen species (ROS) and inflammation-associated immune signaling. Co-administering plant-derived compounds with standard chemotherapeutics may not only reduce off-target toxicity, but also synergistically enhance antitumor responses, suggesting a promising strategy for the design of combination therapies [6,7,8].

Syringic acid (SA), a 3,5-dimethoxyphenolic derivative of gallic acid, is abundantly found in various fruits and vegetables. It exhibits a broad range of biological activities such as antioxidative, anti-inflammatory, antiproliferative, chemoprotective, anti-angiogenic, antidiabetic, antimicrobial, and antidepressant properties [9,10,11]. The anticancer potential of SA has been demonstrated in several cancer types such as colorectal cancer [12,13], lung cancer [14], oral squamous cell carcinoma [15], gastric cancer [16], and ovarian teratocarcinoma [17]. Despite these promising findings, the potential anticancer activity of SA against EC cells has not yet been investigated.

In EC, tumor progression is intimately associated with the modulation of redox signaling and inflammatory pathways within the tumor microenvironment (TME) [18,19,20]. Thus, agents that can selectively modulate oxidative and immune signaling within the TME hold significant potential in improving EC treatment strategies.

This study aimed to investigate the anticancer potential of SA on human endometrioid adenocarcinoma RL95-2 cells, along with its effects on normal human endometrial stromal cells (HESCs), with a focus on cytotoxicity, induction of apoptosis, inhibition of cell migration, modulation of molecular signaling pathways, potential synergistic interactions with chemotherapeutic agents, and the regulation of intracellular inflammatory and antioxidant systems.

## 2. Results

### 2.1. Inhibitory Effects on the Growth of RL95-2 Cells

The effects of SA and four different chemotherapeutic drugs on the growth of RL95-2 cells were analyzed using nuclear fluorescence staining. As shown in Supplementary Figure S1, the intensity of blue fluorescence in the nuclei of RL95-2 cells progressively decreased with increasing drug concentrations, indicating a corresponding reduction in the number of viable cells. Moreover, all tested agents exhibited a dose-dependent decrease in fluorescence intensity, reflecting their cytotoxic effects on RL95-2 cells.

**Figure 1:**
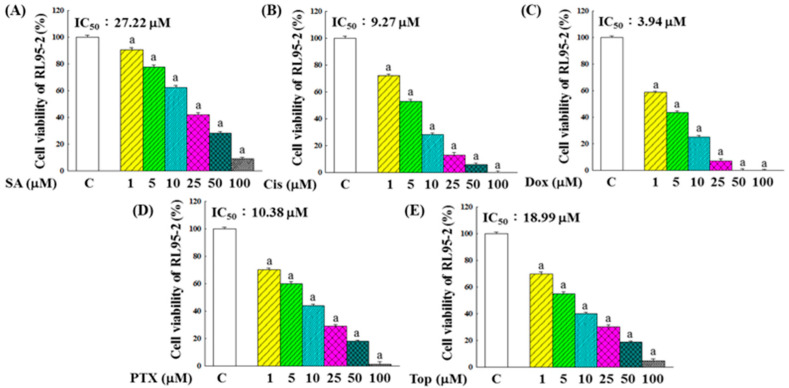
Inhibitory effects of SA (**A**), Cis (**B**), Dox (**C**), PTX (**D**), and Top (**E**) on the proliferation of RL95-2 cells. Following 48 h of drug exposure, cell viability was measured using the MTT assay. Data are presented as the mean ± standard deviation (SD) from five independent experiments. Statistical comparisons between treatment groups and the control (C) group were performed using one-way ANOVA followed by Tukey’s multiple comparisons test. Significant differences (*p* < 0.05) are indicated by “a”.

To precisely quantify the cytotoxicity of these agents, the MTT (3-(4,5-dimethylthiazol-2-yl)-2,5-diphenyltetrazolium bromide) assay was conducted to assess the inhibitory effects of SA and four chemotherapeutic drugs on RL95-2 cell growth. The results, presented in Figure 1, demonstrated a significant reduction in RL95-2 cell viability following treatment with various concentrations of SA or the chemotherapeutic drugs. The IC_50_ values were determined to be 27.22 μM for SA, 9.27 μM for Cis, 3.94 μM for Dox, 10.38 μM for PTX, and 18.99 μM for Top, with Dox exhibiting the highest cytotoxicity against RL95-2 cells.

### 2.2. Synergistic Effects of SA Combined with Anticancer Chemotherapeutic Drugs on the Inhibition of RL95-2 Cell Growth

**Figure 2:**
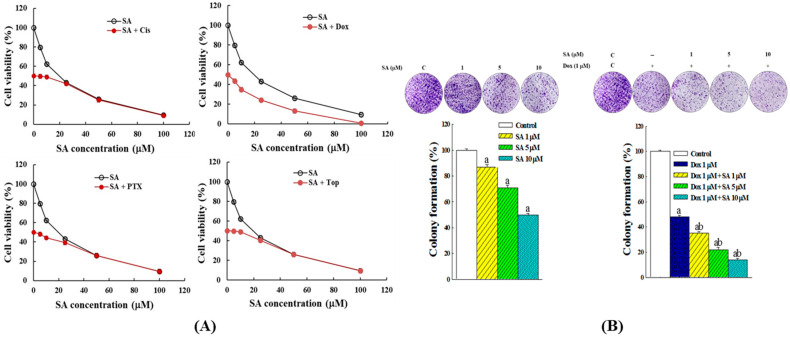
Synergistic suppressive effects of SA in combination with chemotherapeutic agents on RL95-2 cell viability. (**A**) Chemotherapeutic agents were administered at their IC_50_ concentrations, while the dose of SA was varied. After 48 h of co-treatment, cell viability was assessed using the MTT colorimetric assay. (**B**) Effects of SA and the SA-Dox combination on RL95-2 colony formation. Cells were treated with SA at concentrations of 1, 5, or 10 μM, while the SA-Dox combination included a fixed Dox concentration of 1 μM. The culture medium was refreshed every 4 days throughout the incubation period. After 14 days, colonies were stained and quantified. Data represent the results from five independent experimental replicates. Statistical analysis was performed using one-way ANOVA followed by Tukey’s post hoc test. Significant differences were considered at *p* < 0.05, indicated by “a” when compared with the control group and “b” when compared with the vehicle group (Dox alone).

The results presented in Figure 2A demonstrate that the combination of Dox and SA exhibited a significantly stronger synergistic interaction compared with the other three chemotherapeutic agents. The latter showed enhanced cytotoxicity only at lower concentrations of SA, whereas SA concentrations exceeding 20 μM failed to produce additive effects, suggesting that the observed cytotoxicity was primarily mediated by the chemotherapeutic agents alone. Notably, the combination of Dox and SA consistently resulted in lower RL95-2 cell viability across the tested range of SA concentrations when compared with SA treatment alone. Therefore, subsequent investigations focused on the effects of SA administered individually and in combination with Dox.

The colony formation assay was subsequently employed to further assess the synergistic effects of SA and Dox on inhibiting RL95-2 cell growth, using colony numbers as an indicator of viable cell count. As shown in Figure 2B, SA treatment at concentrations of 1, 5, and 10 μM led to a progressive decrease in colony formation, reducing the colony number to 87.2%, 71.6%, and 50.9% of the control, respectively. In contrast, the combination treatment of SA and Dox resulted in a markedly greater inhibition of colony formation, with colony numbers reduced to 35.2%, 22.6%, and 14.3% of the control at the corresponding SA concentrations. These results indicate that the SA-Dox combination exerts a synergistic effect in suppressing RL95-2 cell colony formation.

### 2.3. Expression of Genes Related to Apoptosis and the Cell Cycle

**Figure 3:**
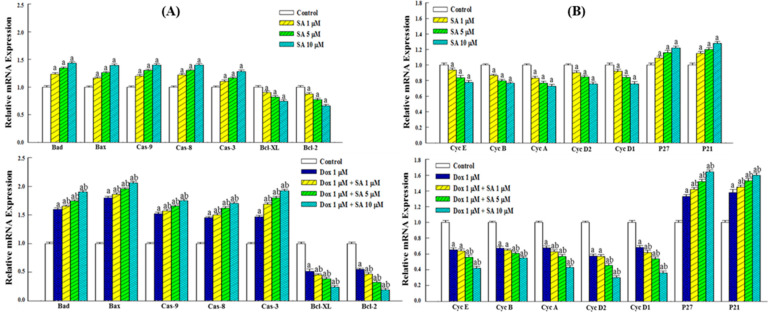
Effects of SA and SA-Dox combination treatments on the expression of genes related to apoptosis (**A**) and the cell cycle (**B**) in RL95-2 cells. Following 48 h of treatment, gene expression levels were measured using RT-PCR. Data represent the means from three independent experiments. Statistical differences between groups were analyzed using one-way ANOVA and Tukey’s multiple comparisons test, with significance defined as *p* < 0.05. Differences compared with the control group are indicated by “a”, and those compared with the vehicle group (Dox alone) are indicated by “b”.

As illustrated in Figure 3A, RT-PCR analysis revealed that SA treatment significantly upregulated the expression of *Caspase-3*, *-8*, and *-9* as well as the pro-apoptotic genes *Bax* and *Bad* in RL95-2 cells. Concurrently, a marked downregulation of the anti-apoptotic genes *Bcl-2* and *Bcl-XL* was observed. These results suggest that SA treatment promotes apoptosis in RL95-2 cells. Treatment with 1 μM Dox alone induced a similar pattern of gene expression, characterized by increased levels of *Caspase-3*, *-8*, *-9*, *Bax*, and *Bad* and decreased levels of *Bcl-2* and *Bcl-XL*, confirming the pro-apoptotic effects of Dox. Moreover, the combination of SA and Dox exerted a more pronounced effect on pro-apoptotic gene expression than either treatment alone, indicating a synergistic enhancement of apoptosis in RL95-2 cells.

As shown in Figure 3B, gene expression analysis demonstrated that SA treatment suppressed the expression of cyclin family genes in RL95-2 cells, suggesting that SA induces cell cycle arrest through cyclin downregulation. Furthermore, SA significantly upregulated the expression of the tumor suppressor genes *p21* and *p27*. These findings support the notion that SA inhibits RL95-2 cell proliferation by downregulating cyclin expression and upregulating *p21* and *p27*. Similarly, treatment with 1 μM Dox alone exerted comparable effects on the expression of these genes. Notably, the combined SA-Dox treatment exhibited a more substantial effect on gene regulation than either agent alone, further indicating a synergistic action in suppressing cancer cell growth.

### 2.4. Inhibitory Effects on RL95-2 Cell Migration

**Figure 4:**
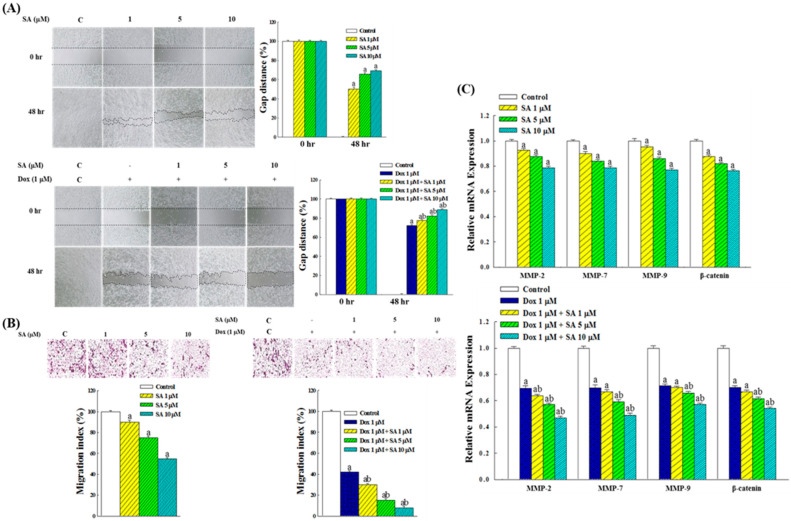
Analysis of the inhibitory effects of SA and SA-Dox combination treatments on RL95-2 cell migration and the underlying mechanisms. (**A**) Wound healing assay was conducted to assess RL95-2 cell motility following treatment with SA and the SA-Dox combination for 48 h. The gap distance, defined as the average distance between two dashed lines in the images, was set at 100% at the initiation of treatment. (**B**) Transwell migration assay was used to quantify the inhibition of RL95-2 cell migration, with cells that migrated to the underside of the membrane stained and counted using a phase-contrast microscope. Migration in the control group was defined as 100%. (**C**) RT-PCR was used to analyze the expression of migration-related genes in RL95-2 cells treated with SA and the SA-Dox combination for 48 h, with the control group values set at 100%. Data were obtained from three independent experimental replicates. Statistical significance between groups was evaluated using one-way ANOVA followed by Tukey’s multiple comparisons test. Differences were considered significant at *p* < 0.05, denoted as “a” when compared with the control group and “b” when compared with the vehicle group (Dox alone).

Wound closure and Transwell migration assays were employed to evaluate the effects of SA and SA-Dox combination treatments on RL95-2 cell migration. The wound closure assay results (Figure 4A) showed that the control group fully closed the wound gap after 48 h of incubation. Treatment with SA (1, 5, and 10 μM) inhibited RL95-2 cell migration, leaving residual gaps corresponding to 50.2%, 65.7%, and 69.3% of the original wound distance, respectively. Dox treatment at 1 μM exhibited a stronger inhibitory effect than 1 μM SA alone. The combination of SA and Dox further enhanced the inhibitory response, with wound gaps remaining at 77.7%, 82.2%, and 89.4% of the original distance, respectively, indicating a synergistic effect in suppressing RL95-2 cell migration.

The results of the Transwell migration assay (Figure 4B) revealed that treatment with SA at 1, 5, and 10 μM for 48 h reduced the migration of RL95-2 cells to 90.2%, 75.4%, and 55.8% of the control, respectively. The inhibitory effect of 1 μM Dox alone was stronger than that of 1 μM SA. Notably, the SA-Dox combination treatment significantly reduced cell migration to 30.3%, 15.2%, and 8.6%, respectively, further highlighting the synergistic inhibitory effect of the combination on RL95-2 cell motility.

### 2.5. Expression of Genes Related to Migration in RL95-2 Cells

Regarding the expression of migration-related genes, Figure 4C shows that SA treatment reduced the expression levels of *MMP-2*, *MMP-7*, *MMP-9*, and *β-catenin* in RL95-2 cells in a dose-dependent manner, indicating that SA effectively suppresses the expression of genes associated with cell migration. Additionally, treatment with 1 μM Dox alone exhibited a stronger inhibitory effect on these genes compared with 1 μM SA. The SA-Dox combination resulted in a more pronounced reduction in the expression of matrix metalloproteinases (*MMP*s) and *β-catenin*, indicating a synergistic interaction between SA and Dox in suppressing the expression of these genes.

### 2.6. Inflammatory Responses in RL95-2 Cells Under SA and Dox Treatments

**Figure 5:**
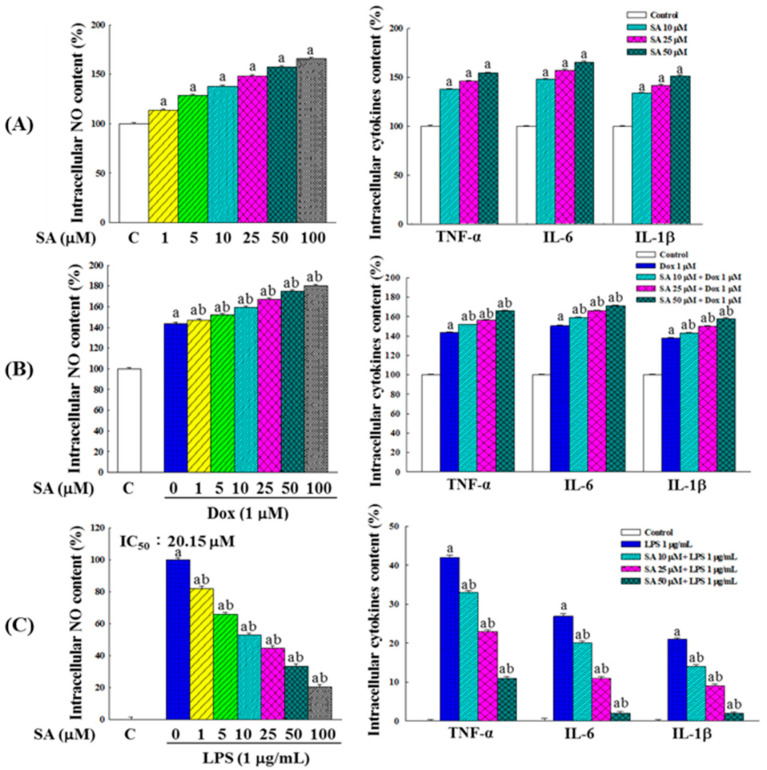
Pro-inflammatory and anti-inflammatory effects of SA treatment on RL95-2 cancer cells and LPS-induced HESC normal cells. (**A**) Effects of various concentrations of SA on intracellular NO, TNF-α, IL-6, and IL-1β levels in RL95-2 cells. (**B**) Effects of Dox and the SA-Dox combination on the same inflammatory markers in RL95-2 cells. (**C**) Effects of different concentrations of SA on the same inflammatory markers in LPS-induced HESC cells. All data were obtained from three independent replicates. Statistical analysis was performed using one-way ANOVA followed by Tukey’s multiple comparisons test. Differences compared with the control group are marked by “a”, while differences relative to the vehicle group (Dox-only or LPS-only) are marked by “b” (*p* < 0.05).

In RL95-2 cells, SA treatment induced an inflammatory response characterized by the increased secretion of NO, TNF-α, IL-6, and IL-1β. The secretion levels positively correlated with the SA concentration (Figure 5A). Compared with 1 μM SA, treatment with 1 μM Dox elicited a stronger inflammatory response. Moreover, the combined treatment with SA and Dox further elevated the NO, TNF-α, IL-6, and IL-1β levels compared with either treatment alone, suggesting an additive effect in promoting inflammation (Figure 5B).

### 2.7. SA Exhibits Anti-Inflammatory and Cytoprotective Effects in HESC Normal Cells

As shown in Figure 5C, HESC cells, which are not inherently inflamed, were treated with lipopolysaccharide (LPS) to induce acute inflammation [21]. SA treatment resulted in a dose-dependent reduction in nitric oxide (NO) production, with an IC_50_ value of 20.15 μM. Similar reductions were observed in the levels of inflammatory cytokines TNF-α, IL-6, and IL-1β, indicating the potent anti-inflammatory properties of SA in normal cells.

**Figure 6:**
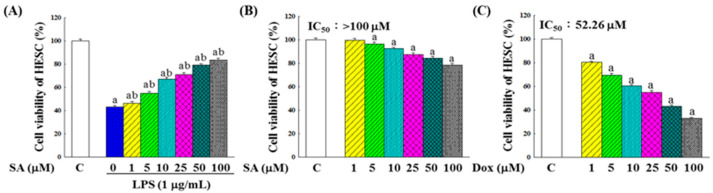
Cytotoxicity of SA and Dox on HESC normal cells. (**A**) Effects of SA on the viability of LPS-stimulated HESC cells. (**B**) Effects of SA on the viability of HESC cells. (**C**) Effects of Dox on the viability of HESC cells. Cell viability was assessed using the MTT assay after 48 h of treatment. Data represent the means of five independent experiments. Statistical analysis was performed using one-way ANOVA followed by Tukey’s multiple comparisons test, with *p* < 0.05 considered statistically significant. Differences compared with the control group are denoted by “a”, and differences compared with the vehicle group (LPS-treated only) are denoted by “b”.

To ensure that the observed decreases in inflammatory markers were not attributable to cell death, cell viability assays were performed. Figure 6A shows that LPS significantly reduced the HESC cell viability, whereas SA treatment improved cell survival in a dose-dependent manner. Additionally, SA exhibited substantially lower cytotoxicity in HESC cells compared with Dox (Figure 6B,C), highlighting its potential to mitigate the adverse effects of chemotherapy.

### 2.8. Effects on the Expression of Inflammation-Related Genes

**Figure 7:**
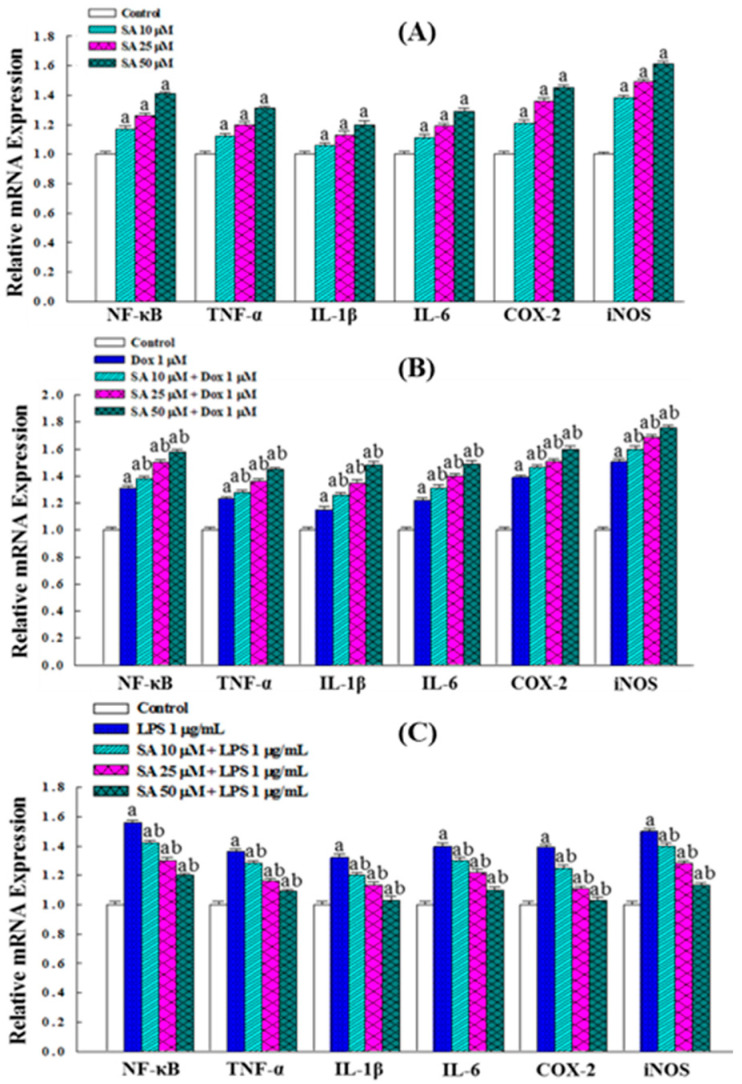
Effects of SA and SA-Dox combination treatments on the expression of inflammation-related genes in RL95-2 cancer cells and HESC normal cells. (**A**) Treatment of RL95-2 cells with various concentrations of SA. (**B**) Treatment of RL95-2 cells with Dox and SA-Dox combination. (**C**) Treatment of LPS-induced HESC cells with various concentrations of SA. Data represent the means of three independent experiments. Statistical significance was determined using one-way ANOVA followed by Tukey’s multiple comparisons test, with *p* < 0.05 considered statistically significant. Differences compared with the control group are denoted by “a”, while differences compared with the vehicle group (Dox- or LPS-treated) are denoted by “b”.

RT-PCR analysis revealed that SA treatment significantly upregulated the expression of *NF-κB*, *TNF-α*, *IL-1β*, *IL-6*, *COX-2*, and *iNOS* (Figure 7A). Treatment with 1 μM Dox alone induced an even higher expression of these genes compared with 10 μM SA. Moreover, combined treatment with SA and Dox for 48 h treatment led to a further significant increase in the expression of these inflammation-related genes, demonstrating an additive effect on their regulation (Figure 7B).

In the LPS-stimulated HESC cells, the expression of these genes was significantly elevated. However, treatment with SA resulted in a dose-dependent decrease in their expression levels as the concentration of SA increased (Figure 7C). This suggests that SA reduces inflammation by inhibiting the expression of cytoplasmic cytokine and suppressing NF-κB activation, thereby preventing NF-κB nuclear translocation and the subsequent induction of COX-2 and iNOS. These effects contribute to the anti-inflammatory and cytoprotective properties of SA.

### 2.9. In Vitro Free Radical Scavenging Activity and Intracellular ROS Production

**Figure 8:**
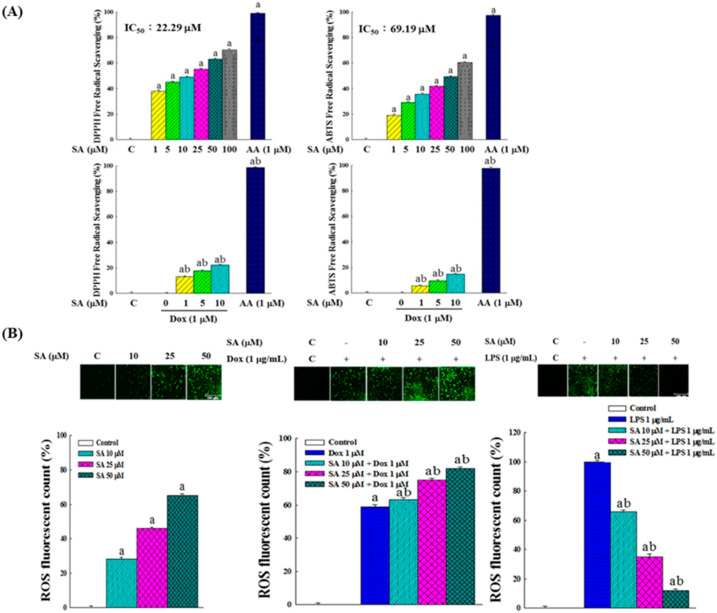
Free radical scavenging capacity and intracellular ROS modulation by SA and Dox. (**A**) Scavenging abilities of SA and SA-Dox combinations against DPPH and ABTS radicals, with ascorbic acid (AA) as the positive control. (**B**) Effects of SA and SA-Dox combinations on intracellular ROS levels in RL95-2 cells and LPS-stimulated HESC cells. Following 48 h of treatment, ROS levels were measured using DCFH2-DA fluorescence intensity. Data represent the means of five independent experiments. Statistical significance was assessed using one-way ANOVA followed by Tukey’s multiple comparisons test, with *p* < 0.05 considered significant. Differences relative to the control group are denoted by “a”, while differences relative to the vehicle group (Dox or LPS alone) are denoted by “b”.

The free radical scavenging activity of SA was evaluated using DPPH (2,2-diphenyl-1-picrylhydrazyl) and ABTS (2,2′-azino-bis(3-ethylbenzothiazoline-6-sulfonic acid)) radicals, as shown in Figure 8A. SA exhibited IC_50_ values of 22.29 μM for DPPH and 69.19 μM for ABTS, demonstrating its effectiveness as an antioxidant. In contrast, Dox showed no radical scavenging ability, and when used in combination with SA, interfered with SA’s activity, thereby reducing SA’s free radical scavenging efficiency.

As illustrated in Figure 8B, both SA and Dox individually increased the intracellular ROS levels in RL95-2 cells. When administered in combination, SA-Dox treatment resulted in a synergistic increase in ROS production, further elevating oxidative stress within the cancer cell microenvironment. This observation suggests a pro-oxidative role of both agents in cancer cells.

In the LPS-induced HESC cells, ROS production was significantly elevated. SA treatment reduced the ROS levels in a dose-dependent manner, suggesting that SA can mitigate LPS-induced oxidative stress. This antioxidant effect underscores the potential of SA to protect normal cells from oxidative damage.

### 2.10. Effects on Intracellular Antioxidant Enzyme Activity and Gene Expression

**Figure 9:**
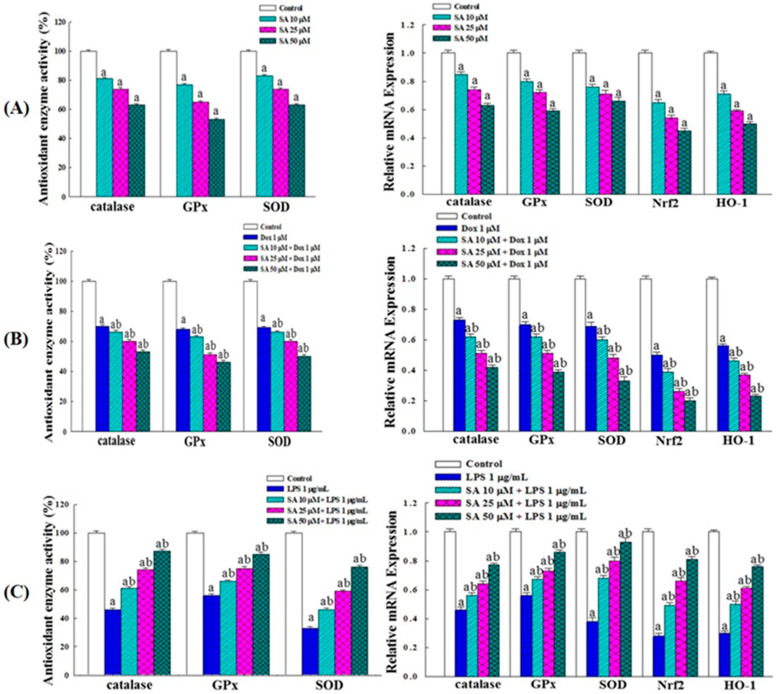
Effects of SA and SA-Dox combination therapy on antioxidant enzyme activities and related gene expression in RL95-2 cancer cells and normal HESC cells. (**A**) Effects of SA on RL95-2 cells. (**B**) Effects of SA-Dox combination therapy on RL95-2 cells. (**C**) Effects of SA on LPS-stimulated HESC normal cells. Cells were cultured for 48 h. Data represent the mean values from three independent experiments. Statistical differences between groups were determined using one-way ANOVA followed by Tukey’s multiple comparisons test, with *p* < 0.05 considered significant. Differences compared with the control group are marked with “a” and those compared with the vehicle group (Dox or LPS alone) are marked with “b”.

In the RL95-2 cells, SA treatment reduced the activities of catalase, glutathione peroxidase (GPx), and superoxide dismutase (SOD) in a dose-dependent manner. Additionally, SA decreased the expression of *catalase*, *GPx*, *SOD*, *HO-1*, and the transcription factor *Nrf2* (Figure 9A). Treatment with Dox alone also reduced these enzyme activities and gene expressions, while the SA-Dox combination further amplified the decline (Figure 9B). These results confirm that SA and Dox suppress antioxidant enzymes and the Nrf2 pathway, thereby increasing oxidative stress to promote cancer cell damage and apoptosis.

In HESC cells, LPS stimulation markedly decreased the activities of catalase, GPx, and SOD as well as the expression of antioxidant-related genes. SA treatment reversed these effects in a dose-dependent manner, significantly enhancing antioxidant enzyme activities (Figure 9C). Treatment with Dox or the SA-Dox combination further increased antioxidant gene expression. These findings indicate that SA and Dox protect normal HESC cells by enhancing antioxidant enzyme activity and Nrf2 pathway expression, thereby reducing oxidative stress and shielding cells from free radical-induced damage.

## 3. Discussion

This study provides new insights into the anticancer potential of SA in the context of EC, specifically focusing on its effects on RL95-2 cancer cells and HESC normal cells. The findings demonstrate that SA not only exhibits anticancer activity, but also enhances the efficacy of the chemotherapeutic agent Dox through multiple mechanisms including apoptosis induction, cell cycle arrest, inhibition of migration, modulation of inflammatory responses, and redox regulation.

One of the major findings of this study was the dose-dependent cytotoxicity of SA in RL95-2 cells, which was comparable to that of conventional chemotherapeutic agents (Figure 1). Notably, the SA-Dox combination exhibited a significantly enhanced effect on reducing cell viability and colony formation (Figure 2), suggesting synergistic cytotoxicity. This enhancement was supported by the upregulation of *Caspase-3*, *-8*, *-9*, *Bax*, and *Bad* and the downregulation of the anti-apoptotic genes *Bcl-2* and *Bcl-XL* (Figure 3A). These molecular events indicate that SA facilitates programmed cell death through multiple converging pathways, and when combined with Dox, the apoptotic signaling cascade is further amplified.

In addition to apoptosis, SA induced a strong antiproliferative effect by modulating key regulators of the cell cycle. SA treatment significantly downregulated *cyclin D1*, *D2*, *E*, *A*, and *B* expression, and upregulated the cyclin-dependent kinase (CDK) inhibitors *p21* and *p27* (Figure 3B), which are known to induce G1/S and G2/M arrest. These results suggest that SA effectively suppresses EC cell proliferation by interfering with cyclin-CDK complexes and enforcing checkpoint control. Moreover, the SA-Dox combination reinforced these effects, providing a mechanistic basis for the observed synergistic inhibition of cell growth.

Another critical aspect of EC progression is metastasis, which involves complex processes such as extracellular matrix (ECM) degradation and epithelial–mesenchymal transition (EMT) [22,23]. The present study showed that SA inhibited RL95-2 cell migration in the wound healing and Transwell assays (Figure 4A,B) by downregulating the expression of matrix metalloproteinases (*MMP-2*, *-7*, and *-9*) and *β-catenin* (Figure 4C). Since these markers are key mediators of ECM remodeling and EMT, their suppression may attenuate the invasive, metastatic, and angiogenetic potential of EC cells [22]. Furthermore, β-catenin is a central component of the Wnt signaling pathway, modulating the transcription of several downstream genes such as *MMP*s, *c-Myc*, *cyclin D1*, and *vimentin*, which has been implicated in cancer progression and therapeutic resistance [24,25,26]. Therefore, the inhibitory effect of SA on β-catenin expression may have broader implications for disrupting oncogenic signaling and improving treatment outcomes. The stronger inhibitory effect observed with the SA-Dox combination underscores its potential to mitigate metastasis, recurrence, and chemoresistance in EC, ultimately improving patient outcomes.

The dual role of SA in modulating inflammation was also investigated. In cancer cells, both SA and Dox increased pro-inflammatory cytokines (TNF-α, IL-6, IL-1β) and NO, likely contributing to an acute inflammatory response that facilitates immunogenic cell death (Figure 5A,B). Consistently, gene expression analysis revealed the upregulation of *NF-κB*, *TNF-α*, *IL-1β*, *IL-6*, *COX-2*, and *iNOS* (Figure 7A,B), indicating the activation of inflammatory signaling pathways. While inflammation is often associated with tumor progression, acute inflammatory responses may also promote cancer cell death via immune activation [27]. The heightened inflammatory response observed with the SA-Dox combination suggests a possible mechanism for enhancing tumor immunogenicity and therapeutic efficacy.

In contrast, in the HESC normal cells exposed to LPS-induced acute inflammation, SA exerted a protective anti-inflammatory effect. SA treatment significantly reduced the NO and cytokine levels and downregulated inflammation-related gene expression (Figure 5C and Figure 7C). Additionally, SA preserved cell viability in LPS-treated HESC cells (Figure 6A), suggesting that it mitigates inflammation-induced cytotoxicity. This dual function, pro-inflammatory in cancer cells and anti-inflammatory in normal cells, highlights SA’s selective modulation of the TME, which may be crucial for enhancing the efficacy and safety margin of chemotherapy [28].

Redox regulation is another important mechanism through which SA exerts its effects. Although SA displayed strong in vitro antioxidant activity with IC_50_ values of 22.29 μM and 69.19 μM for DPPH and ABTS radicals, respectively (Figure 8A), it induced pro-oxidative stress in RL95-2 cells by elevating intracellular ROS levels (Figure 8B). This increase was accompanied by the reduced activity of catalase, GPx, and SOD as well as the downregulation of Nrf2 signaling (Figure 9A,B), indicating suppression of the antioxidant defense system. These changes likely contributed to ROS-mediated apoptosis and cancer cell damage [29,30]. Importantly, the SA-Dox combination further amplified ROS accumulation, providing a plausible mechanism for their synergistic cytotoxicity.

In contrast, SA demonstrated antioxidant properties in HESC cells, where it reversed LPS-induced oxidative stress by restoring antioxidant enzyme activities and upregulating Nrf2-related genes (Figure 9C). These effects suggest that SA selectively enhances oxidative stress in cancer cells while protecting normal cells from ROS-mediated injury, which is a desirable characteristic for adjuvant agents in chemotherapy. This dichotomy may involve context-dependent redox signaling and immunomodulatory pathways such as ROS- and endoplasmic reticulum (ER) stress-induced immunogenic cell death [31], which merit further exploration.

The study’s findings also raise important translational considerations. Among the chemotherapeutic agents tested, Dox and SA both act via ROS accumulation and oxidative stress pathways [32,33]. This shared mechanism may account for their synergistic effects; however, it also raises concerns about potential off-target toxicity, particularly cardiotoxicity associated with Dox. Previous studies have linked Dox-induced cardiac injury to mitochondrial dysfunction and calcium dysregulation [34,35,36]. Using SA to reduce the required Dox dose may offer a strategy to minimize these adverse effects. However, this hypothesis needs to be tested in vivo, with comprehensive assessments of cardiac safety and therapeutic efficacy.

Despite the promising findings, this study had several limitations. All experiments were conducted in vitro, and the lack of animal models limits the ability to predict in vivo responses and clinical relevance. Furthermore, while the study investigated key pathways related to apoptosis, cell cycle regulation, inflammation, and oxidative stress, other mechanisms, such as autophagy, ER stress, immune cell recruitment, or angiogenesis, were not evaluated. Future research should address these gaps using in vivo models to assess the pharmacokinetics, bioavailability, and systemic toxicity. Different delivery routes, such as oral, intraperitoneal, or local administration, should also be compared to optimize the therapeutic outcomes. Toxicological evaluations, including organ-specific toxicity and potential drug–drug interactions, will be essential before clinical application.

**Figure 10:**
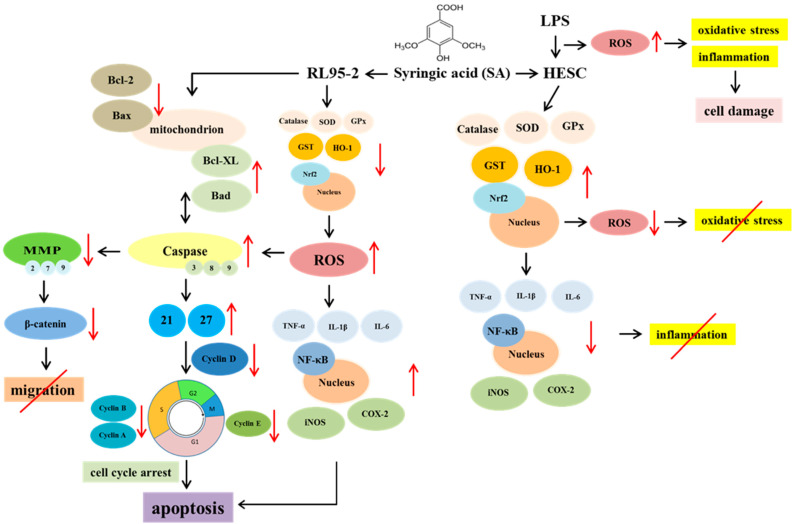
Proposed mechanism of action of SA in RL95-2 EC cells and HESC normal cells. SA inhibits the proliferation and migration of RL95-2 cells by activating caspase cascades, modulating Bcl-2 family proteins, causing cell cycle arrest via the upregulation of *p21* and *p27*, suppressing *MMP-2*, *-7*, *-9*, and *β-catenin*, and inducing ROS accumulation and acute inflammation. In parallel, SA reduces LPS-induced oxidative stress and inflammation in HESC normal cells by activating the Nrf2 antioxidant pathway and downregulating NF-κB-mediated pro-inflammatory responses, highlighting its dual role in anticancer activity and cytoprotection. The black arrows indicate the direction of progression. The upward and downward red arrows represent upregulation and downregulation, respectively. The red diagonal slash denotes that the effect is mitigated.

In summary, this study demonstrates that SA exerts multifaceted anticancer effects in RL95-2 EC cells while providing cytoprotective benefits in HESC normal cells (Figure 10). When combined with Dox, SA enhances anticancer efficacy through the synergistic modulation of apoptosis, cell cycle arrest, migration inhibition, inflammation, and oxidative stress. These findings support the potential of SA as an adjuvant therapeutic agent in EC, warranting further preclinical investigation to facilitate clinical translation.

## 4. Materials and Methods

### 4.1. Cell Cultivation

RL95-2, a human uterine endometrial adenocarcinoma cell line, and the HESC cell line were obtained from the Bioresource Collection and Research Center (Hsinchu, Taiwan). Both cell lines were cultured in a CO_2_ incubator at 37 °C using DMEM/F12 medium supplemented with 10% fetal bovine serum (FBS) (Thermo Fisher Gibco, Waltham, MA, USA), 100 units/mL penicillin, 100 μg/mL streptomycin, and 2 mM L-glutamine to maintain optimal growth conditions.

### 4.2. Nuclear Fluorescence Staining

The proliferation of RL95-2 cells was assessed using nuclear fluorescence staining. Cells were dispensed at a density of 5 × 10^4^ cells per well in a 96-well plate and treated with serial concentrations of the experimental compounds for 48 h. Following incubation, the supernatant was aspirated, and cells were fixed with 500 μL of 4% formaldehyde (Merck Sigma-Aldrich, Rahway, NJ, USA) for 10 min. Subsequent membrane permeabilization was achieved using 500 μL of 0.5% Triton X-100 at 4 °C for 5 min. Nuclear staining was carried out by adding 100 μL of 0.2% Hoechst 33342 (Merck Sigma-Aldrich) for 5 min, and fluorescence microscopy was employed for visualization and quantitative analysis.

### 4.3. Cell Viability Assay

Cell viability was assessed utilizing the MTT reduction assay (Merck Sigma-Aldrich). Following a 48 h incubation period in culture media containing varying concentrations of the test compounds, the supernatant was discarded. Subsequently, cells were treated with 100 μL of DMEM/F12 medium supplemented with 5 mg/mL MTT reagent for 2 h. The absorbance of solubilized formazan product was measured at 570 nm using an ELISA microplate reader. The half-maximal inhibitory concentration (IC_50_) was defined as the compound concentration required to suppress 50% of cancer cell proliferation.

### 4.4. Clonogenic Survival Assay

RL95-2 cells were seeded at a density of 1 × 10^3^ cells per well in a 6-well plate and allowed to establish adherence for 24 h. Subsequently, the cells were exposed to the test samples, and the culture medium was replaced every four days. After 14 days, the resulting colonies were fixed using 1 mL of Carnoy’s solution (Merck Sigma-Aldrich). Then, the cells were rinsed with phosphate-buffered saline (PBS) and stained with 1 mL of 10% Giemsa solution (Merck Sigma-Aldrich) for 3 min. The extent of colony formation was evaluated using phase-contrast microscopy.

### 4.5. Wound Healing Assay

RL95-2 cells (1 × 10^7^) were cultivated in a medium containing a culture insert (Merck Sigma-Aldrich) for 24 h to allow for the formation of a monolayer. The culture insert was then carefully removed to create a defined cell-free gap, followed by incubation with varying concentrations of test agents for 48 h. Cellular migration across the denuded region was visualized and documented using an inverted phase-contrast microscope (Nikon Eclipse TS100, Tokyo, Japan).

### 4.6. Transwell Migration Assay

Cell suspensions containing 1 × 10^7^ cells and varying concentrations of test samples were loaded into the upper compartment of a Boyden chamber insert from the CytoSelect™ Cell Migration Assay Kit (Cell Biolabs, San Diego, CA, USA). The lower chamber was filled with serum-containing medium to provide a chemoattractant gradient. After 48 h of incubation, non-migratory cells on the upper membrane surface were removed, and the insert was immersed in methanol for 20 min to fix the migrated cells. Subsequently, adherent cells were immersed in 400 μL of crystal violet staining solution (Merck Sigma-Aldrich) for 30 min, followed by air-drying and microscopic visualization of migratory capability.

### 4.7. Intracellular NO Analysis

A freshly prepared Griess reagent was obtained by combining a 1% sulfanilamide solution with a 0.1% N-1-naphthylethylenediamine dihydrochloride solution (Merck Sigma-Aldrich) in a 1:1 ratio. Cells were treated with various concentrations of the test samples, with or without 1 μg/mL LPS, for 48 h to stimulate an inflammatory response. Following the incubation, 80 μL of the collected supernatant was combined with 80 μL of Griess reagent and incubated in the dark for 10 min. The optical density was then determined at 540 nm using an ELISA reader.

### 4.8. Intracellular Cytokine Analysis

Intracellular levels of TNF-α, IL-6, and IL-1β were quantified using commercial ELISA kits (Merck Sigma-Aldrich). The procedure involved the overnight coating of 96-well microplates with capture antibodies. After washing with buffer, assay diluent was introduced, followed by a 1 h incubation at room temperature. Subsequently, 100 μL of culture supernatant was added and allowed to react for 2 h. After additional washing steps, 100 μL of working detector solution was added and incubated for 1 h. After a final washing step, 50 μL of substrate solution was introduced and allowed to react for 30 min, followed by the addition of 50 μL of stop solution to halt the reaction. Absorbance was measured at 450 nm for cytokine quantification.

### 4.9. Free Radical Scavenging Capacity Assay

To evaluate the antioxidant potential of the samples, spectrophotometric analysis was performed using two radical probes, DPPH and ABTS (MCE, Monmouth, NJ, USA). For the DPPH radical scavenging assay, 100 μL of the sample solution was mixed with 25 μL of 0.5 mM DPPH reagent, followed by incubation at room temperature in the dark for 30 min. The optical density change was measured at 517 nm to determine the extent of DPPH radical scavenging.

For the ABTS radical scavenging assay, a stable ABTS radical solution was generated by mixing 7.4 mM ABTS solution with 2.6 mM potassium persulfate in equal volumes, followed by a 12 h incubation in the dark at room temperature. Subsequently, 20 μL of the sample solution was added to 180 μL of the ABTS solution and incubated at room temperature for 2 h. The optical density change at 410 nm was measured to determine the ABTS radical scavenging capacity.

### 4.10. Intracellular ROS Analysis

Intracellular ROS levels were quantified using the Fluorometric Intracellular ROS Kit (Merck Sigma-Aldrich). Cells subjected to 48 h incubation were thoroughly rinsed with phosphate-buffered saline and exposed to 100 μL of a 5 μM 2′,7′-dichlorofluorescin diacetate solution for 1 h. Fluorescence intensity, indicative of ROS levels, was measured using a BioTek microplate reader (Synergy™ 2, Winooski, VT, USA) with an excitation wavelength of 502 nm and an emission wavelength of 524 nm.

### 4.11. Antioxidant Enzyme Activity Assay

Cells cultured for 48 h were subjected to protein extraction using a cell lysis buffer. The protein concentration in the cell lysate was determined using the BCA Protein Assay Kit (Merck Sigma-Aldrich). Subsequently, the activities of key antioxidant enzymes, including catalase, GPx, and SOD, were quantified using commercial enzyme activity assay kits (Merck Sigma-Aldrich).

### 4.12. Gene Expression Assay

Cells designated for gene expression analysis underwent total RNA extraction using the Qiagen RNeasy Kit (Qiagen, Venlo, The Netherlands). Complementary DNA (cDNA) was synthesized by reverse transcription of the extracted RNA using the Magic RT cDNA Synthesis Kit (Bio-Genesis, Taipei, Taiwan). Subsequently, qPCR amplification of the synthesized cDNA was performed using the IQ2 SYBR Green Fast qPCR Synthesis Master Mix LOW ROX Kit (Bio-Genesis) on a 7500 Fast Dx Real-Time PCR System (Applied Biosystems, Foster City, CA, USA). The primers used in the amplification reactions are listed in Table 1. The thermal cycling parameters consisted of three stages: an initial incubation at 50 °C for 2 min, polymerase activation at 95 °C for 10 min, followed by 40 cycles of denaturation at 95 °C for 15 s and annealing/elongation at 60 °C for 1 min.

### 4.13. Statistical Analyses

Experimental replicates consisted of three to five independent trials, and the resulting data were represented as mean values with corresponding standard deviations (±SD). Variability across experimental groups was examined using a one-way analysis of variance (ANOVA). When significant differences were detected, Tukey’s multiple comparisons test was applied for post hoc pairwise analysis. Statistical analyses were performed using SPSS version 25.0 (IBM, Armonk, NY, USA) with a significance criterion set at *p* < 0.05.

## Figures and Tables

**Table 1:** Primer sequences utilized in the RT-PCR assay.

Gene	Forward (5′−3′)	Reverse (5′−3′)
*GAPDH*	TGCACCACCAACTGCTTAGC	GGCATGGACTGTGGTCATGAG
*Bad*	CAGTGATCTGCTCCACATTC	TCCAGCTAGGATGATAGGAC
*Bax*	CGGCGAATTGGAGATGAACTGG	CTAGCAAAGTAGAAGAGGGCAACC
*Caspase-9*	TCAGTGACGTCTGTGTTCAGGAGA	TTGTTGATGATGAGGCAGTAGCCG
*Caspase-8*	ACAAGGGCATCATCTATGGCTCTGA	CCAGTGAAGTAAGAGGTCAGCTCAT
*Caspase-3*	GTGGAACTGACGATGATATGGC	CGCAAAGTGACTGGATGAACC
*Bcl-xL*	AACATCCCAGCTTCACATAACCCC	GCGACCCCAGTTTACTCCATCC
*Bcl-2*	CTGAGTACCTGAACCGGCA	GAGAAATCAAACAGAGGCCG
*Cyclin E*	GGAAGAGGAAGGCAAACG	GCAATAATCCGAGGCTTG
*Cyclin B*	AAGGTGCCTGTGTGTGAACC	GTCAGCCCCATCATCTGCG
*Cyclin A*	AACGATGAGCACGTCCCTAC	CAGCTGGCCTCTTCTGAGTC
*Cyclin D2*	TGTGGATTGTCTCAAAGCCTG	CAACATCCCGCACGTCTGTA
*Cyclin D1*	CAGAAGTGCGAAGAGGAGGTC	TCATCTTAGAGGCCACGAACAT
*p27*	GGTGCCTTCAATTGGGTCTC	GCTTCCTCATCCCTGGACAC
*p21*	GAGCAGTGCCCGAGTTAAGG	TGGAACAGGTCGGACATCAC
*MMP-2*	AGAACTTCCGATTATCCCATGATGA	TGACAGGTCCCAGTGTTGGTG
*MMP-7*	GGCGGAGATGCTCACTTTGAC	AATTCATGGGTGGCAGCAAAC
*MMP-9*	GCCCTGGAACTCACACGACA	TTGGAAACTCACACGCCAGAAG
*β-Catenin*	ATTGATTCGAAACCTTGCCC	AGCTCCAGTACACCCTTCTA
*NF-κB*	GAAATTCCTGATCCAGACAAAAAC	ATCACTTCAATGGCCTCTGTGTAG
*TNF-α*	CAGGTTCTGTCCCTTTCACTCACT	GTTCAGTAGACAGAAGAGCGTGGT
*IL-1β*	GGTCAAAGGTTTGGAAGCAG	TGTGAAATGCCACCTTTTGA
*IL-6*	TGGAGTACCATAGCTACCTGGAGT	TCCTTAGCCACTCCTTCTGTGACT
*COX-2*	CCGGGTACAATCGCACTTAT	GGCGCTCAGCCATACAG
*iNOS*	CTCAGCCCAACAATACAAGATGACCCTAAG	AGAGTGAGCTGGTAGGTTCCTGTTGTTTCT
*Cat*	GCCATTGCCACAGGAAAGTA	CCTTGGTGAGATCGAATGGA
*GPx*	CCAAGCTCATCACCTGGTCT	TCGATGTCAATGGTCTGGAA
*SOD*	TGGCCGATGTGTCTATTGAA	CACCTTTGCCCAAGTCATCT
*Nrf2*	CAGCGACGGAAAGAGTATGA	TGGGCAACCTGGGAGTAG
*HO-1*	CATGACACCAAGGACCAGAG	AGTGTAAGGACCCATCGGAG

## Data Availability

Data is contained within the article.

## Supplementary Materials

The following supporting information can be downloaded at: https://www.mdpi.com/article/10.3390/ph18111596/s1, Supplementary Figure S1. Analysis of RL95-2 cell growth inhibition by varying concentrations of SA, Cis, Dox, PTX, and Top using nuclear fluorescence staining. Cells were treated with the indicated agents for 48 h. (A) Representative images of Hoechst 33342-stained nuclei. (B) Quantitative analysis of nuclear fluorescence intensity based on five independent biological replicates. Statistical analysis was performed using one-way ANOVA followed by Tukey’s multiple comparisons test. Significant differences compared with the untreated control (C) group are indicated by “a” (*p* < 0.05).

## Abbreviations

The following abbreviations are used in this manuscript:

ABTS	2,2′-Azino-bis(3-ethylbenzothiazoline-6-sulfonic acid)
ANOVA	One-way analysis of variance
cDNA	Complementary DNA
Cis	Cisplatin
DPPH	2,2-Diphenyl-1-picrylhydrazyl
Dox	Doxorubicin
EC	Endometrial cancer
ECM	Extracellular matrix
EMT	Epithelial–mesenchymal transition
ER	Endoplasmic reticulum
FBS	Fetal bovine serum
GPx	Glutathione peroxidase
HESC	Human endometrial stromal cells
LPS	Lipopolysaccharide
MMPs	Matrix metalloproteinases
MTT	(3-(4,5-Dimethylthiazol-2-yl)-2,5-diphenyltetrazolium bromide
NO	Nitric oxides
PBS	Phosphate-buffered saline
PGE2	Prostaglandin E2
PTX	Paclitaxel
ROS	Reactive oxygen species
RT-PCR	Real-time polymerase chain reaction
SA	Syringic acid
SD	Standard deviation
SOD	Superoxide dismutase
TME	Tumor microenvironment
Top	Topotecan
