# Supplementary material for: Inhibitory Effects of Syringic Acid on Endometrial Cancer Cell Growth and Migration and Its Synergistic Suppression with Doxorubicin
Source: Pharmaceuticals (Basel). 2025 Oct 22;18(11):1596. doi: 10.3390/ph18111596 (PMC12655531; doi:10.3390/ph18111596)
Supplement: Supplementary file 1 [file pharmaceuticals-18-01596-s001.zip › pharmaceuticals-3749069-supplementary.pdf]

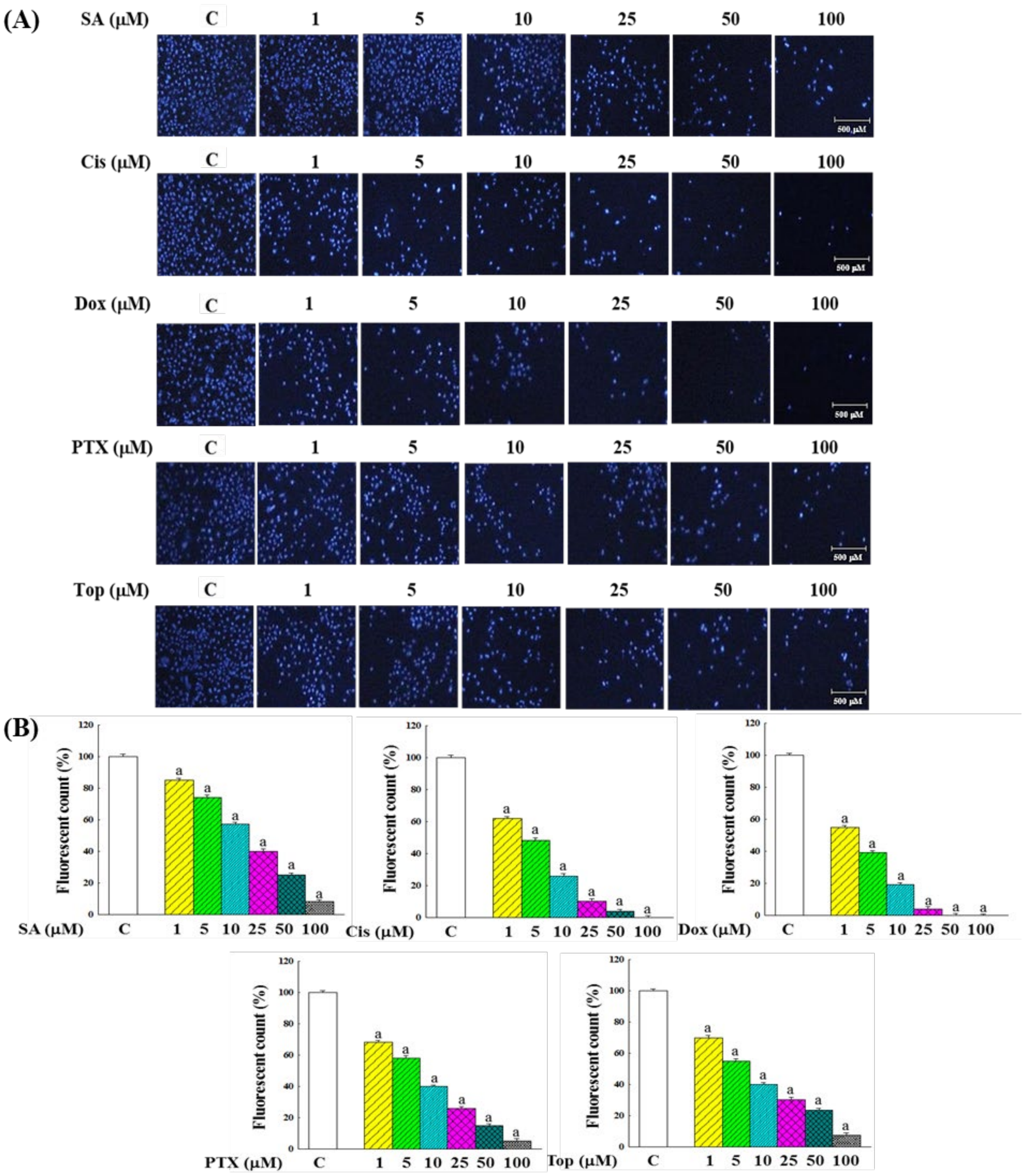

Supplementary Figure S1. Analysis of RL95-2 cell growth inhibition by varying concentrations of SA, Cis, Dox, PTX, and Top using nuclear fluorescence staining. Cells were treated with the indicated agents for 48 h. (A) Representative images of Hoechst 33342-stained nuclei; (B) Quantitative analysis of nuclear fluorescence intensity based on five independent biological replicates. Statistical analysis was performed using one-way ANOVA followed by Tukey's multiple comparisons test. Significant differences compared to the untreated control (C) group are indicated by "a" ($p < 0.05$).
